# Interleukin-1β and transforming growth factor-β cooperate to induce neurosphere formation and increase tumorigenicity of adherent LN-229 glioma cells

**DOI:** 10.1186/scrt96

**Published:** 2012-02-10

**Authors:** Lei Wang, Ziyan Liu, Sivasai Balivada, Tej Shrestha, Stefan Bossmann, Marla Pyle, Loretta Pappan, Jishu Shi, Deryl Troyer

**Affiliations:** 1 Department of Anatomy and Physiology, College of Veterinary Medicine, Kansas State University, Manhattan, KS 66506, USA; 2 Department of Chemistry, College of Arts and Sciences, Kansas State University, Manhattan, KS 66506, USA

## Abstract

**Introduction:**

Glioma stem cells (GSCs) have the property of self-renewal and appear to be a driving force for the initiation and recurrence of gliomas. We recently found that the human tumorigenic LN-229 glioma cell line failed to form neurospheres in serum-free conditions and generated mostly small tumors *in vivo*, suggesting that either LN-229 GSCs are not active in these conditions or GSCs are absent in the LN-229 cell line.

**Methods:**

Using self-renewal assay, soft-agar colony assay, cell proliferation assay, invasion assay, real time PCR analysis, ELISA and *in vivo *tumorigenic assay, we investigated the effects of interleukin (IL)-1β and transforming growth factor (TGF)-β on the development of GSCs from LN-229 cells.

**Results:**

Here, we demonstrate that the combination of IL-1β and TGF-β can induce LN-229 cells to form neurospheres in serum-free medium. IL-1β/TGF-β-induced neurospheres display up-regulated expression of stemness factor genes (nestin, Bmi-1, Notch-2 and LIF), and increased invasiveness, drug resistance and tumor growth *in vivo*: hallmarks of GSCs. These results indicate that IL-1β and TGF-β cooperate to induce a GSC phenotype in the LN-229 cell line. Induction of nestin, LIF and Notch-2 by IL-1β/TGF-β can be reverted after cytokine withdrawal. Remarkably, however, up-regulated Bmi-1 levels remained unchanged after cytokine withdrawal; and the cytokine-withdrawn cells maintained strong clonogenicity, suggesting that Bmi-1 may play a crucial role in tumorigenesis.

**Conclusions:**

Our finding indicates that glioma cells without self-renewal capability in standard conditions could also contribute to glioma malignancy when cytokines, such as IL-1β and TGF-β, are present in the tumor environment. Targeting GSC-promoting cytokines that are highly expressed in glioblastomas may contribute to the development of more effective glioma therapies.

## Introduction

Gliomas are the most common primary brain tumors in adults and glioblastomas are the most malignant gliomas (WHO grade IV). Despite substantial progress in early diagnosis, surgery and radiation protocols, only 15% of patients who undergo radical surgery are still alive within two years after diagnosis [1]. The poor prognosis is mainly attributed to the highly diffusive growth pattern of glioblastoma cells into surrounding brain tissue, preventing complete surgical removal. Although there has been intensive effort devoted to understanding the molecular mechanisms involved in the genesis and progression of glioma, effective treatment of this tumor type still remains elusive [2,3].

Cancer stem cells (CSCs) or tumor-initiating cells (TICs) are a subpopulation of tumor cells with the ability to undergo self-renewal and recapitulate the entire tumor population [4]. Similar to CSCs from other types of cancers, glioma stem cells (GSCs) have been identified from human glioma tissues and glioma cell lines by applying conditions typically used for isolation of neural stem cells (NSCs) [5-9]. GSCs are characterized by the ability of self-renewal to generate spheres termed "neurospheres" or "glioma neurospheres" when cultured in serum-free conditions supplemented with epidermal growth factor (EGF) and basic fibroblast growth factor (bFGF). These glioma neurospheres reflect biological and pathological characteristics of primary gliomas, display resistance to chemo- and radiotherapies, and, more importantly, have enhanced oncogenic potential, generating tumors that reproduce the characteristics of the original tumors after intracranial transplantation [8,10,11]. Thus, understanding the biology of GSCs may provide therapeutic strategies for the better treatment of glioma by targeting GSCs.

Self-renewal of GSCs appears to be a driving force for "successful" tumor growth, and significant effort has been made to elucidate its regulatory mechanisms [12]. In recent years, the *in vitro *neurosphere formation assay in serum-free conditions has been established as a measure of GSC self-renewal [13]. This assay has been validated by use of xenotransplantation models that are considered as the gold standard for evaluation of GSC self-renewal and tumor initiation [6]. Accordingly, the ability to form neurospheres *in vitro *is defined as an important aspect of GSCs [14], and has been employed to verify GSCs that are isolated from gliomas using varied methods, as well as to determine molecular factors that regulate self-renewal of GSCs [15,16].

Inflammatory cytokines play a key role in malignant progression of various cancer types [17]. In human glioblastomas, pro-inflammatory cytokines, interleukin (IL)-1, IL-6 and IL-8, are expressed and secreted at high levels, and their expression levels are correlated with the histological grade of the neoplasms [18-21]. The essential role of IL-6 in glioma development has been demonstrated in a mouse model [22]. Transforming growth factor-β (TGF-β), an immunosuppressive cytokine, is also significantly elevated in high-grade gliomas and increased TGF-β activity confers poor prognosis in glioma patients [23,24]. It has been suggested that the inflammatory cytokines contribute to tumor malignancy by promoting invasion, metastasis, angiogenesis and evasion from immune attack [17,25,26]. Interestingly, these cellular behaviors promoted by cytokines are also characteristics of GSCs [27], suggesting that cytokines may contribute to the development of GSCs. Indeed, recent findings suggest that cytokine signaling pathways play a critical role in the regulation of GSC self-renewal. For example, higher expression levels of IL-6 receptors have been found in GSCs than non-stem glioma cells, and targeting IL-6 ligand or receptor expression in GSCs significantly reduced neurosphere formation and tumor growth [16]. TGF-β signaling has also been shown to increase self-renewal capacity and prevent differentiation of GSCs [28-31].

However, these studies focus on a rare population of GSCs that can actively divide in serum-free medium to elucidate self-renewal regulatory mechanisms. Recent data show that not all GSCs can generate neurospheres, suggesting that there are different subtypes of GSCs and some GSCs remain inactive in standard conditions [32]. In addition, differentiated glioma cells have been reported to reprogram towards a stem cell phenotype in hypoxia and acidic conditions, suggesting that GSC can be a dynamic stage because of cancer cell plasticity [33,34]. Taken together, these studies implicate that microenvironment conditions can regulate GSC activities and development. Recently, we found that the human glioma cell line LN-229 failed to form neurospheres in serum-free conditions, suggesting that GSCs in LN-229 are not active in these conditions or they are absent in this line. Therefore, in this study, we used the LN-229 cell model to investigate the effects of IL-1β and TGF-β on development of GSCs.

## Materials and methods

### Cell culture

Human glioma cell lines U87, T98, U138, and D54 were obtained from the American Type Culture Collection (Manassas, VA, USA). LN-229 was a kind gift from Dr. Gilbert Cote (MD Anderson Cancer Center, Houston, TX, USA). All cells were maintained in Dulbecco's modified Eagle's medium (DMEM) with 10% fetal bovine serum (FBS) and 1% penicillin-streptomycin (Invitrogen Corp., Carlsbad, CA, USA) in a humidified incubator with 37°C and 5% CO_2_. For neurosphere induction, cells were cultured in serum-free medium (SFM), which consisted of neurobasal-A medium supplemented with B27, GlutaMAX-I supplement, 1% penicillin-streptomycin (Invitrogen Corp.), 50 ng/ml heparin (Sigma-Aldrich, St. Louis, MO, USA), 20 ng/ml of EGF, and 20 ng/ml bFGF (R&D Systems, Minneapolis, MN, USA). To induce neurosphere formation of LN-229 cells, 200 pM IL-1β and 200 pM TGF-β1 (both from R&D Systems) were added every other day to the serum-free medium. IL-6 and IL-8 (both from R&D Systems) were also used to evaluate their effects on induction of neurospheres.

### Self-renewal assay and cell proliferation assay

LN-229 cells at a clonal density of 1 cell/μl in serum-free medium were seeded at 100 μl/well in 96-well plates and treated with or without 200 pM IL-1β and/or 200 pM TGF-β1 for seven days. IL-1β and TGF-β1 were added every other day. The total number of neurospheres in each well was counted under a microscope. Cell proliferation was measured using the cell proliferation kit I (MTT, Roche Applied Science, Indianapolis, IN, USA) as described by the manufacturer.

### RNA extraction and RT-PCR

Total RNA was extracted using TRI reagent (Sigma-Aldrich), followed by digestion with a DNase kit (Applied Biosystems, Carlsbad, CA, USA) to remove DNA residues. Reverse transcription was carried out using the iScript cDNA synthesis kit (Bio-Rad, Hercules, CA, USA) and quantitative real-time PCR was performed using SsoFast Eva Green Supermix kit (Bio-Rad).

### Immunostaining and immunoblotting

LN-229 cells were seeded in slide chambers (Fisher Scientific, Hanover Park, IL, USA) and cultured for seven days in serum-free medium in the absence or presence of IL-1β/TGF-β. Then, cells were fixed with 4% paraformaldehyde, permeablized with PBS containing 0.2% Triton X-100, and incubated with mouse anti-nestin (1:50; Santa Cruz Biotechnology, Santa Cruz, CA, USA), and followed by secondary chicken anti-mouse IgG (H+L) antibody conjugated with Alexa 488 (1:200, Invitrogen). Cells were then mounted with VECTASHIELD Mounting Medium with DAPI (Vector laboratories, Burlingame, CA, USA) and observed with a confocal microscope.

LN-229 cells were cultured in SFM in the absence or presence of IL-1β/TGF-β for seven days. Cells were then washed with cold PBS, lysed in RIPA buffer (25 mM Tris-HCl (pH 7.6), 150 mM NaCl, 1% NP-40, 1% sodium deoxycholate, 0.1% SDS) and pelleted by centrifugation. Protein concentrations were determined using a NanoDrop instrument (Thermo Scientific, Wilmington, DE, USA). Cell lysates (30 μg protein for each sample) were incubated for five minutes at 100°C in 2x loading buffer, separated by electrophoresis in 10% polyacrylamide gels, and transferred to PVDF membranes (Millipore, Bedford, MA, USA). Membranes were blocked with 5% milk in PBS and then incubated with primary antibody anti-Bmi-1 clone F6 (1:1,000 dilution, Millipore), or anti-β-actin (1:1,000 dilution, Sigma), and secondary antibody HRP-conjugated goat anti-mouse IgG-HRP (1:1,000 dilution, Millipore) or anti-rabbit IgG HRP-linked antibody (1:1,000 dilution, Cell Signaling, Danvers, MA, USA), respectively. Detection was performed using HyGLO substrate (Denville Scientific, Metuchen, NJ, USA) and images were taken using the AlphaEaseFC imaging system (Cell Biosciences, Santa Clara, CA, USA).

### Invasion assay

The invasion assay was performed using 24-well Matrigel invasion chambers (BD Biosciences, San Jose, CA, USA). Single control monolayer cells and IL-1β/TGF-β-induced neurosphere cells were resuspended in serum-free medium and loaded into the top inserts (2.5 × 10^4^/500 μl). DMEM with 20% FBS was added to the lower chamber (750 μl) as a chemoattractant. After incubation for 48 h at 37°C, non-invasive cells were removed from the top of the matrigel with a cotton tipped swab. Invasive cells that migrated through 8-μm pores to the underside of the membrane were fixed with 100% methanol and stained with 0.005% crystal violet before counting under a microscope.

### Chemoresistance assay

Control monolayer and IL-1β/TGF-β-induced neurosphere cells in serum-free medium were treated with temozolomide (Sigma-Aldrich) at a concentration of 250, 500, 1,000 μM for two days. Then cells were stained with Trypan blue (Amresco Inc., Solon, OH, USA) and counted under a microscope. The viability of the cells was measured by the percentage of live cells over the total of live and dead cells.

### Soft-agar colony-forming assay

The soft agar assay was performed in six-well plates containing two layers of Sea Plague Agar (Invitrogen). The bottom layer consisted of 0.8% agar in 1 ml of DMEM with 10% FBS. Control monolayer and IL-1β/TGF-β-induced neurosphere cells were dissociated and placed (1 × 10^4^/well) in the top layer containing 0.4% agar in the same medium as the bottom. Cells were cultured for two to three weeks and colonies were photographed under a microscope and measured using the ImageJ program (http://imagej.nih.gov). Colonies with diameters larger than 80 μm were counted.

### Transfection of LN-229 cells with Gaussia luciferase

LN-229 cells were transfected with a pCMV-*G*luc-1 vector encoding the *Gaussia *luciferase gene (Nanolight Technology, Pinetop, AZ, USA) using Lipofectamine 2000 (Invitrogen). The stable transfectants were selected with gentamicin-418 at 1,000 μg/ml. Expression of *G*luc was confirmed by a luciferase assay with the IVIS Lumina II imaging system (Caliper Life Sciences, Hopkinton, MA, USA).

### Implantation of LN-229 cells into immunocompromised mice

The LN-229 cells stably expressing *G*luc were used for an *in vivo *study. Athymic nude mice (nu/nu, six to seven weeks old; Charles River Laboratories, Wilmington, MA, USA) were stereotactically implanted with the control monolayer cells (3 × 10^5 ^cells/mouse) or IL-1β/TGF-β-induced neurosphere cells (3 × 10^5 ^cells/mouse) in 3 μl of PBS into the right striatum (1 mm anterior and 2 mm lateral to bregma; 3.5 mm intraparenchymal). Five weeks after implantation, animals were subjected to bioluminescence imaging, followed by euthanization. Brains were removed, placed in Tissue-Tek O.C.T. compound (Sakura Finetek, Torrance, CA, USA), and quickly frozen in liquid nitrogen. The frozen tissues were then stored at -80°C until sectioning as described below. All animal procedures were approved by the Institutional Animal Care and Use Committee (IACUC) of Kansas State University and the IACUC number is 2813.

### Bioluminescence imaging

Bioluminescence imaging was performed using the IVIS Lumina II imaging system (Caliper Life Sciences). To determine the correlation between the number of *G*luc-expressing cells and the bioluminescence signal *in vitro*, various numbers of the monolayer or IL-1β/TGF-β-induced neurosphere cells were used, and images were taken one minute after a substrate for *G*luc (60 μg/ml coelenterazine) was added to the serum-free medium. For *in vivo *bioluminescence imaging, coelenterazine (150 μg/animal in 150 μl of PBS) was given i.v.; animals were anesthetized with 2.5 to 3% isoflurane for approximately two minutes and then placed inside the camera box (Caliper Life Sciences) that maintained 2% isoflurane for the duration of imaging. The imaging scan time for each animal was one minute.

### Coelenterazine synthesis and solution preparation

Coelenterazine was synthesized by an extensive modification of the procedure reported by Adamczyk *et al. *[35]. Aminopyrazine was used as a starting material to generate 3,5-diboromoaminopyrizine as a building block for introducing the pyrazine moiety of coelenterazine. A series of Negishi coupling reactions was used to cross-couple the benzyl and 4-hydroxyphenyl groups to 3,5-diboromoaminopyrizine by using Huo's and Buchwald's approach to zincation [36,37]. The complete synthesis and characterization of coelentearzine will be published separately.

Coelenterazine and hydroxypropyl-beta-cyclodextrin (Acros Organics, Pittsburgh, PA, USA) (1:50, w:w) was dissolved in methanol (ACS grade). Methanol was removed by high vacuum distillation to obtain a dry yellow powder of the mixture of coelenterazine and cyclodextrin. This mixture was dissolved in autoclaved distilled water prior to imaging. A total of 150 μL of coelentrazine (1 mg/mL coelenterazine), which is the substrate for *Gaussia *luciferase, was injected i.v. prior to tumor imaging.

### Brain sectioning, H&E staining and tumor volume calculation

Mouse brains were cryostat-cut at 10 μm thickness in coronal sections, mounted on Probe One Plus slides (Fisher Scientific) and stained with an H&E stain kit (BBC Biochemical, Stanwood, WA, USA).

### Enzyme-linked immunosorbent assay (ELISA)

Cells were treated with or without IL-1β and TGF-β in serum-free medium for six days. Cells were then washed with PBS and cultured in serum-free medium without cytokines for 24 h at 37°C. Cell supernatants were harvested and assayed for IL-1β and IL-8 using Quantikine ELISA Kits (R&D Systems) as described by the manufacturer.

### Statistical analysis

Student's *t-*test was used to determine statistical significance for all analyzed data except the *in vivo *bioluminescence signal experiment. A Fisher's exact test was used to compare the percentage of mice with positive bioluminescence signals. In all cases, we consider a two-sided *P *< 0.05 significant.

## Results

### The combination of IL-1β and TGF-β induced neurosphere formation of LN-229 cells in serum-free medium

**Figure 1:**
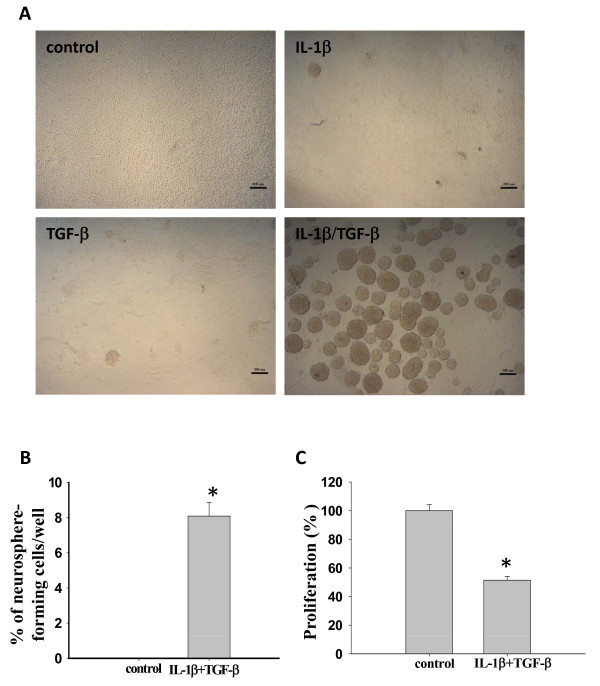
**The combination of IL-1β and TGF-β induces neurosphere formation of LN-229 cells**. **(A**) Representative images of LN-229 cells in the absence or presence of IL-β and TGF-β. LN-229 cells (10,000 cells/well) were cultured in six-well plates containing serum-free medium (SFM) in the absence or presence of 200 pM IL-1β or/and 200 pM TGF-β for seven days. Scale bar = 200 μm. (**B**-**C**) Neurosphere formation and proliferation of LN-229 cells cultured at a clonal density (1 cell/μl) in 96-well plates containing 100 μl SFM in each well with or without IL-1β/TGF-β for 14 days. (**B**) The percentage of newly formed neurospheres per well in the presence IL-1β/TGF-β. *P < 0.05. (**C**) IL-1β/TGF-β-induced neurosphere cells proliferated more slowly than control cells, as demonstrated using an MTT assay. Error bars represent SEM. *P < 0.05.

**Figure 2:**
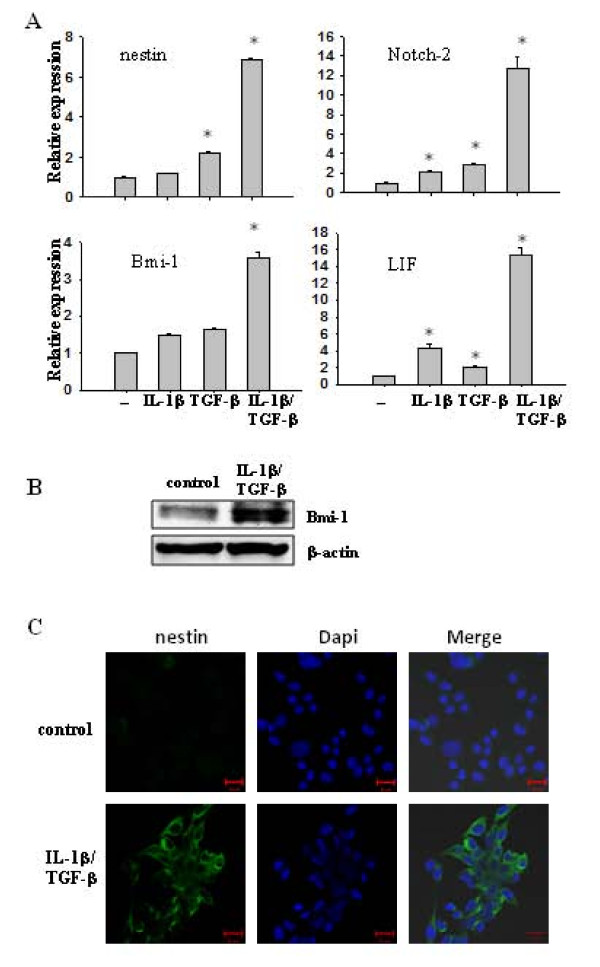
**IL-1β/TGF-β-induced neurosphere cells express stem cell markers**. **(A**) Transcription levels of stemness genes in control and IL-1β/TGF-β-induced neurospheres. LN-229 cells were cultured in SFM with or without IL-1β/TGF-β for seven days. Differential mRNA levels of stem cell markers were determined by qRT-PCR. β-actin was used as an internal normalization control. Error bars represent SEM. *P < 0.01. (**B**) Immunoblot analysis for Bmi-1 on cell lysates from control monolayer cells and IL-1β/TGF-β-induced neurospheres. β-actin was used as an internal normalization control. **(C**) IL-1β/TGF-β-induced neurospheres express nestin, as demonstrated using an immunostaining assay. LN-229 cells were cultured in SFM with or without IL-1β/TGF-β for seven days and stained with anti-nestin antibody. Nuclei (blue stain) were counterstained with DAPI. Scale bar = 20 μm.

In a preliminary study of GSCs, we compared the growth properties of human glioblastoma cell lines U87, T98, U138, D54 and LN-229 in serum-free medium supplemented with EGF and bFGF. A single cell line, LN-229, did not grow as neurospheres, but monolayer cells under these conditions (Figure 1A). Immunocytochemical analysis showed that the monolayer culture of LN-229 cells had low level expression of nestin (a well-known neural stem/progenitor cell marker) (Figure 2C). The lack of neurosphere formation and low nestin expression of LN-229 culture in serum-free medium suggests that GSCs are absent in the LN-229 cell line or they are inactive in this condition.

IL-1β and TGF-β are highly expressed in malignant gliomas and associated with poor prognosis of glioma patients. Therefore, we hypothesized that TGF-β and IL-1β may contribute to GSC growth. To test the hypothesis, we investigated whether IL-1β and TGF-β could induce neurosphere formation of LN-229 cells. IL-1β, TGF-β, or the combination of both cytokines was added to LN-229 cell culture in serum-free medium. As shown in Figure 1A, very few clusters were formed in the presence of IL-1β or TGF-β alone and most cells remained as a monolayer in the seven-day cultures. However, in the presence of IL-1β and TGF-β, LN-229 cells proliferated as clusters at first, which then grew bigger and detached from the bottom of plates to form neurospheres. The neurosphere formation induced by IL-1β/TGF-β, but not IL-1β or TGF-β alone, indicates a synergistic effect of IL-1β and TGF-β on the induction of neurospheres.

To avoid cell aggregation that leads to neurosphere formation, LN-229 cells were plated in 96-well plates at a clonal density of 1 cell/μl in serum-free medium in the presence or absence of IL-1β and TGF-β. As shown in Figure 1B, 8% of the LN-229 cells treated with IL-1β/TGF-β formed neurospheres. Meanwhile, we compared the proliferation rates of LN-229 monolayer cells and IL-1β/TGF-β-induced neurospheres using an MTT assay. As shown in Figure 1C, neurospheres induced by IL-1β/TGF-β grew significantly (*P *< 0.05) more slowly than the untreated monolayer cells.

### IL-1β/TGF-β-induced neurosphere cells express stem cell markers

To further characterize IL-1β/TGF-β-induced neurospheres and determine the molecular mechanisms underlying these observations, we evaluated the expression of several stemness genes in LN-229 cells treated with or without IL-1β, TGF-β, or the combination of both in serum-free medium. Using real time PCR, we found that the expression of nestin, Notch2. Bmi-1 and LIF was up-regulated significantly in IL-1β/TGF-β-induced LN-229 neurospheres, while they were slightly increased in cells treated with IL-1β or TGF-β alone, compared to control cells (Figure 2A). The enhanced expression of Bmi-1 and nestin in IL-1β/TGF-β-induced neurospheres was further confirmed by immunoblotting and immunofluoresence staining, respectively (Figure 2B, C). These results indicate that the pathways of IL-1β and TGF-β signaling interact to augment the expression of stemness genes, leading to the formation of neurospheres (Figure 1A).

### IL-1β/TGF-β-induced neurospheres of LN-229 cells displayed augmented drug resistance and cell migration

**Figure 3:**
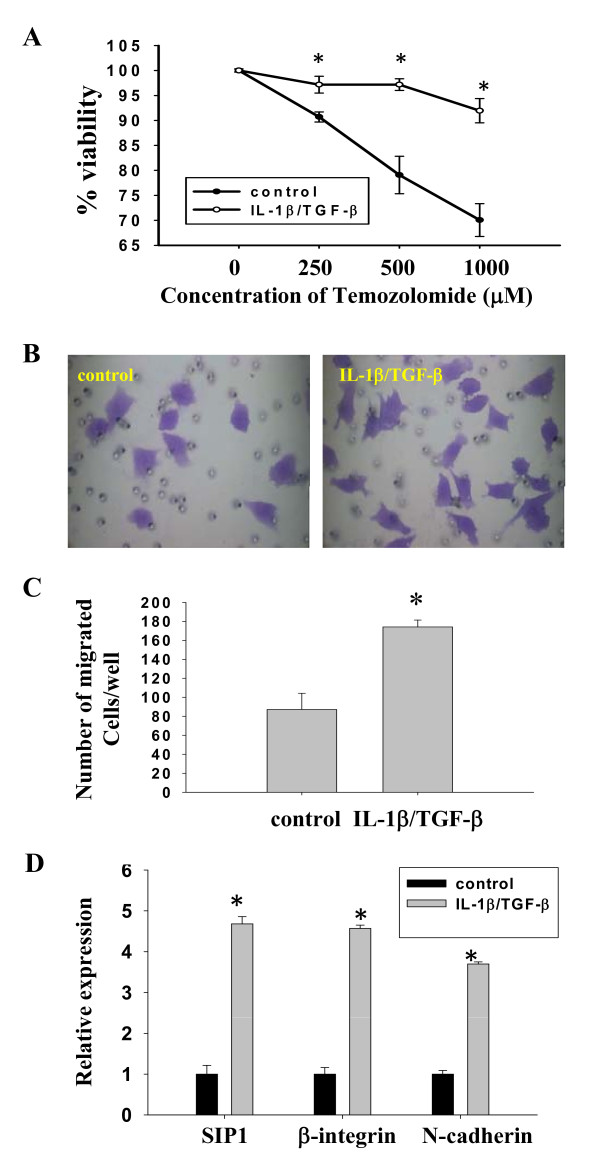
**IL-1β/TGF-β-induced neurosphere cells show enhanced drug resistance and invasiveness**. (**A**) IL-1β/TGF-β-induced neurosphere cells develop drug resistance. The control and IL-1β/TGF-β-induced neurosphere cells were treated with various concentrations of temozolomide for two days. Then, cells were dissociated and stained with Trypan blue, and counted under a microscope. The viability was determined by the percentage of live cells over the sum of live and dead cells. *P < 0.01. (**B, C, D**) IL-1β/TGF-β-induced neurosphere cells have stronger invasiveness and enhanced invasive gene expression. LN-229 cells were cultured in SFM in the absence or presence of IL-1β/TGF-β for seven days and then the control monolayer cells and IL-1β/TGF-β-induced neurosphere cells were dissociated. 5 × 10^4 ^cells of each group were placed on the top well of a Matrigel Invasion chamber. DMEM medium with 20% FBS was added to the lower chamber. After 48 h of incubation, non-invaded cells were removed from the top chamber and invaded cells at the lower surface were fixed and stained with 0.005% crystal violet. Representative images of migrated cells were shown in **(B)**. Migrated cells/well were counted under a microscope and reported in **(C)**. *P < 0.01. (**D**) Transcription levels of invasive genes in control monolayer and IL-1β/TGF-β-induced neurosphere cells. LN-229 cells were cultured in SFM in the absence or presence of IL-1β/TGF-β for seven days and relative gene expression was determined by qRT-PCR analysis. β-actin was used as an internal normalization control. Error bars represent SEM. *P < 0.005.

To determine whether IL-1β/TGF-β-induced neurospheres possessed typical GSC properties, such as drug resistance and invasiveness, we first compared the cytotoxic effect of temozolomide, a chemotherapeutic agent for human glioblastomas, on control monolayer LN-229 cells and IL-1β/TGF-β-induced neurospheres. Control LN-229 and neurosphere cells were treated with various concentrations of temozolomide for two days. Cell viability was measured as the percentage of live cells relative to total cell number (dead + live cells). As shown in Figure 3A, IL-1β/TGF-β-induced neurospheres displayed significantly (*P *< 0.01) greater resistance to temozolomide at concentrations range from 250 to 1,000 μM.

We then evaluated the invasiveness of IL-1β/TGF-β-induced neurospheres using a transwell migration assay. Equal numbers of dissociated single cells from IL-1β/TGF-β-induced neurospheres and control monolayer LN-229 cultures were placed on transwell inserts. Invasiveness was measured by counting the cells that had migrated through the insert 48 h post inoculation (Figure 3B). It was found that twice as many of IL-1β/TGF-β-induced neurosphere cells migrated through the insert compared to that of control cells (Figure 3C).

To understand the molecular mechanisms underlying the enhanced migration, we examined the gene expression profile of several proteins that are known to be involved in cell migration and invasion. It was found that the expression of Smad interacting protein 1 [SIP1; also known as zinc finger binding protein 2 (ZEB2)], β-integrin and neuronal (N)-cadherin were significantly (*P *< 0.005) increased in IL-1β/TGF-β-induced neurosphere cells compared to that in control LN-229 cells (Figure 3D). This result suggests that SIP1, β-integrin, and N-cadherin might be involved in the migration of IL-1β/TGF-β-induced neurosphere cells.

### IL-1β/TGF-β-induced neurospheres displayed increased oncogenic potential *in vitro *and *in vivo*

**Figure 4:**
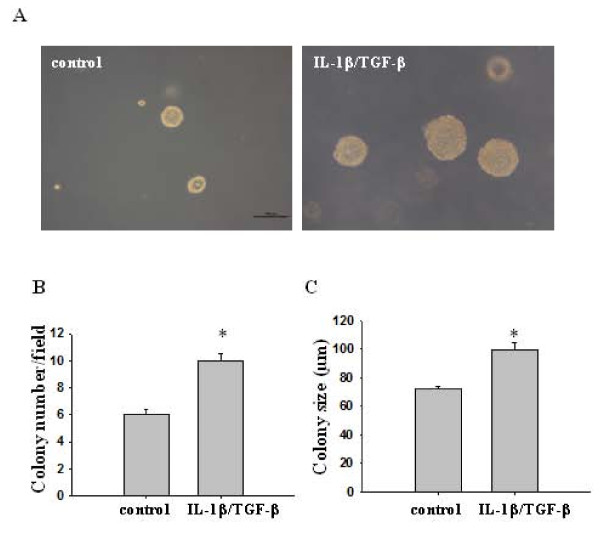
**IL-1β/TGF-β-treated cells form more and larger colonies than control cells in soft agar**. Single control or IL-1β/TGF-β-treated cells were plated in soft agar containing DMEM and 10% FBS in six-well plates at a density of 10,000 cells/well and cultured for 14 days. (**A**) Representative images of the colonies derived from the control and IL-1β/TGF-β-induced neurosphere cells. Scale bar = 100 μm. Colony number was counted under a microscope as shown in (**B**) and size was measured using ImageJ as shown in **(C)**. Twenty random fields were selected for measurement and the data represent the average colony number and size per microscope field. *P < 0.001.

To assess the oncogenic potential of IL-1β/TGF-β-induced neurospheres, we performed a colony-forming assay that is frequently utilized for *in vitro *evaluation of malignant transformation and tumorigenesis [31]. Cytokine-induced neurospheres and control monolayer LN-229 cells were disassociated and then seeded in soft agar at the same concentration. The total number and size of colonies formed in soft agar wells were determined after the cells were cultured for 14 days. As shown in Figure 4, IL-1β/TGF-β-induced neurosphere cells generated significantly more (Figure 4B) as well as larger (Figure 4C) colonies than the control monolayer cells, indicating that IL-1β/TGF-β-induced neurosphere cells possessed higher oncogenic potential.

**Figure 5:**
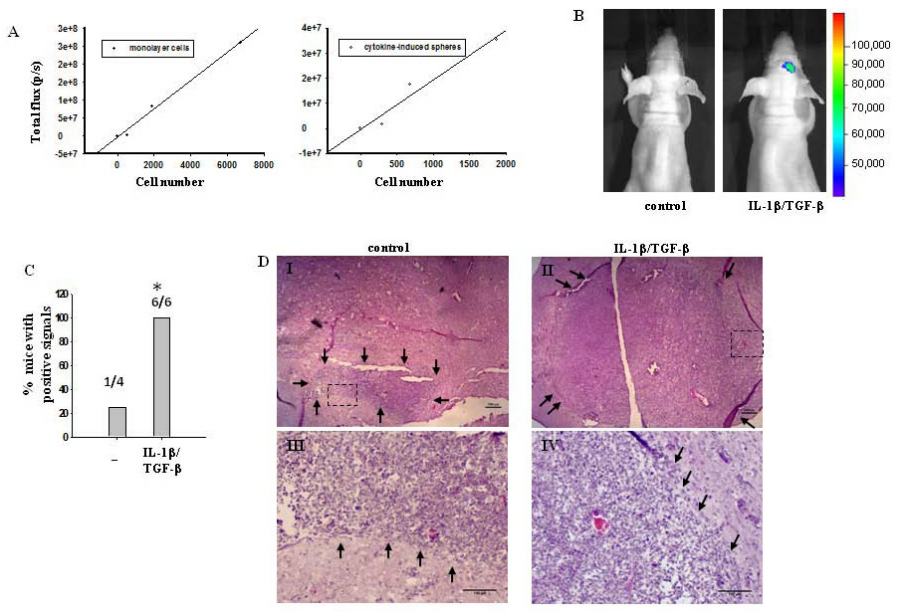
**IL-1β/TGF-β-induced neurosphere cells have increased tumorigenic potential**. (**A**) Bioluminescence characteristics of LN-229 cells. Various numbers of LN-229 cells expressing *Gaussia *luciferase were cultured in 96-well plates in SFM in the absence or presence of IL-1β/TGF-β for seven days, then incubated with 60 μg/ml coelenterazine and imaged with a scan time of one minute. After imaging, cells were counted under a microscope. The relationship between bioluminescent signal strength and cell number for control monolayer LN-229 cells (left panel) and for IL-1β/TGF-β-induced neurosphere cells (right panel) was reported here. (**B**) Representative images of mice injected intracranially with control or IL-1β/TGF-β-induced neurosphere cells. 3 × 10^5 ^cells of the control monolayer or IL-1β/TGF-β-induced neurosphere cells expressing *Gaussia *luciferase were injected into each nu/nu mouse brain at 1 mm anterior, 2 mm from bregma to right lateral and 3.5 mm intraparenchymal. Images were taken five weeks after cell injection, two minutes after administering coelenterazine i.v. with a scan time of one minute. (**C**) The percentage of mice which had at least three times higher bioluminescent signal at the injection site than the background signal. *P < 0.05. (**D**) H&E staining of cryosectioned mouse brain five weeks after injection with control monolayer cells or IL-1β/TGF-β-induced neurosphere cells. Small black arrows demarcate tumor margin. (**III) **and (**IV**) Amplified images of the areas indicated by rectangles in (**I) **and (**II**), respectively. Scale bars in (**I) **and (**II**) = 200 μm. Scale bars in (**III) **and (**IV**) = 100 μm.

To further evaluate the oncogenic potential of IL-1β/TGF-β-induced neurosphere cells, we examined whether the cytokine-induced neurosphere cells can form tumors *in vivo*. Bioluminescent imaging, which has been recently established to monitor and quantify *in vivo *tumor growth and cell survival [38,39], was used in our study to track brain tumor development from injected LN-229 cells transfected with a vector expressing *Gaussia *luciferase (*G*luc). LN-229 cells stably expressing *G*luc were established and cultured in serum-free medium in the absence or presence of IL-1β/TGF-β for seven days. The relationship between bioluminescent signal strength and cell number was determined using various numbers of cells from either control monolayer cells or IL-1β/TGF-β-induced neurospheres. It was found that bioluminescent signals generated from both populations had a strong positive linear correlation with cell number within the range tested (Figure 5A). However, control monolayer LN-229 cells displayed a higher level of *G*luc activity than cytokine-induced neurospheres. This finding is consistent with the report that luciferase expression is higher in monolayer tumor cells than in tumor neurospheres [40].

In the present study, mice were injected with control monolayer cells or IL-1β/TGF-β-induced neurosphere cells (3 × 10^5^/mouse) intracranially. Five weeks after implantation, tumor growth was evaluated using an IVIS imaging system that measures luciferase expression level within the brain. A positive signal for tumor growth was recognized when the signal detected at the injection site was at least three-fold of that at a non-injection site (that is, background) on each mouse. Based on this standard, it was found that one out of four mice bearing the control monolayer cells and six out of six mice bearing the IL-1β/TGF-β-induced neurosphere cells showed positive signals for tumor growth (Figure 5B, C). This result suggests that IL-1β/TGF-β-induced neurosphere cells have significantly (*P *< 0.03) greater capability of generating tumors that can be detected by a bioluminescence imaging system. This difference is more pronounced if one considers that monolayer LN229 cells expressed higher amount of *G*luc than did the cytokine-induced sphere cells (Figure 5A).

To verify the *in vivo *tumor growth, brains were collected from animals on Day 35 post implantation and processed for H&E staining as described in Materials and methods. Figure 5D shows the representative images of middle sections of tumors from mice bearing IL-1β/TGF-β-induced neurosphere cells or control monolayer LN-229 cells with negative bioluminescence signals. Although tumors were detected by H&E staining at the injection site in all mice, the failure to detect a bioluminescence signal in tumors from mice implanted with the control monolayer LN-229 cells suggests that the sensitivity of this bioluminescence imaging system is limited.

### IL-1β/TGF-β-induced neurosphere cells maintained higher levels of Bmi-1 gene expression and oncogenic potential after cytokine removal

**Figure 6:**
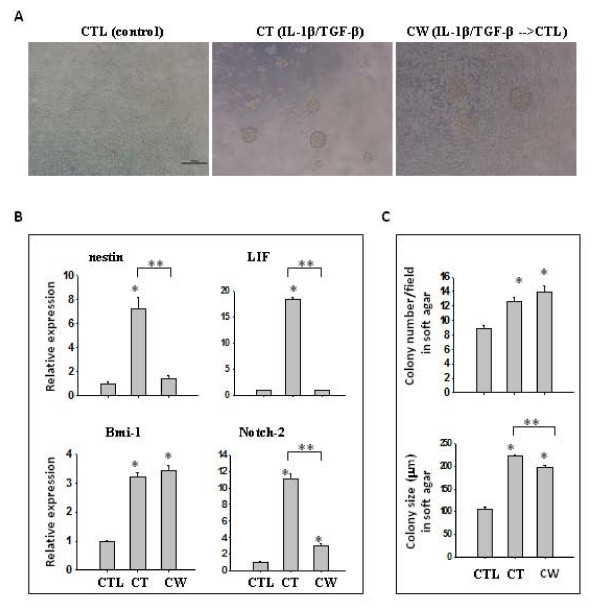
**Cytokine-withdrawn cells after initial IL-1β/TGF-β exposure retain stem-like properties**. **(A**) Representative images of LN-229 cells cultured in SFM in the absence (CTL, (control)) or presence of IL-1β/TGF-β (CT, (IL-1β/TGF-β)), or after withdrawal of both cytokines following initial exposure (CW, (IL-1β/TGF-β → CTL)) for seven days. Scale bar = 200 μm. **(B**) qRT-PCR analysis of gene expression of stem cell markers in CTL, CT and CW cells. β-actin was used as an internal normalization control. (**C**) The number and size of colonies formed in soft agar. CTL, CT and CW cells were dissociated and 10,000 cells/well of each population were cultured in soft agar in six-well plates for 21 days. Colony number was counted under a microscope and colony size was measured using ImageJ. Twenty random fields were selected for the measurement and the data represent the average colony number and size per microscope field. *P < 0.05. **P < 0.01. Error bars represent SEM.

To determine whether IL-1β/TGF-β-induced neurosphere cells still maintained self-renewal ability to form neurospheres in the absence of these two cytokines, neurospheres were dissociated into single cells and cultured in serum-free medium without IL-1β and TGF-β for seven days. The cytokine-withdrawn (CW) cells grew mostly as monolayer cells with some clusters, a mixed morphology of control (CTL, no IL-1β and TGF-β) and continued cytokine-treated (CT) cells (Figure 6A). Expression of stemness genes, including nestin, LIF, Bmi-1 and Notch-2 in these three types of LN-229 cells was analyzed using real time PCR. As shown in Figure 6B, expression of nestin and LIF in cytokine-treated cells was augmented as reported earlier (Figure 2A), but their expression in cytokine-withdrawn cells was reduced to the same level as control cells. Although Notch-2 expression was dramatically reduced in cytokine-withdrawn cells compared to that in cytokine-treated cells, it was still significantly (*P *< 0.001) higher than that in control LN-229 cells. However, the key finding was that the expression of Bmi-1 in cytokine-withdrawn cells remained as elevated as that in cytokine-treated cells (Figure 6B).

The oncogenic potential of cytokine-withdrawn cells was evaluated by a colony forming assay. Control, cytokine-treated and cytokine-withdrawn cells were dissociated into single cells. Then, an equal number of cells from each group were cultured in soft agar and colonies were counted after 21 days. Consistent with previous results, cytokine-treated cells generated significantly (*P *< 0.005) more and larger colonies than control LN-229 cells (Figure 6C). Interestingly, cytokine-withdrawn cells produced a similar number of colonies as cytokine-treated cells but with a slightly reduced size. However, their size was still significantly (*P *< 0.004) larger than that of colonies generated by control cells (nearly two-fold, Figure 6C). This result indicates that the molecules responsible for the augmented *in vitro *oncogenic potential remained active in cytokine-withdrawn cells.

### IL-8 might be involved in IL-1β/TGF-β-mediated induction of LN-229 neurospheres

**Figure 7:**
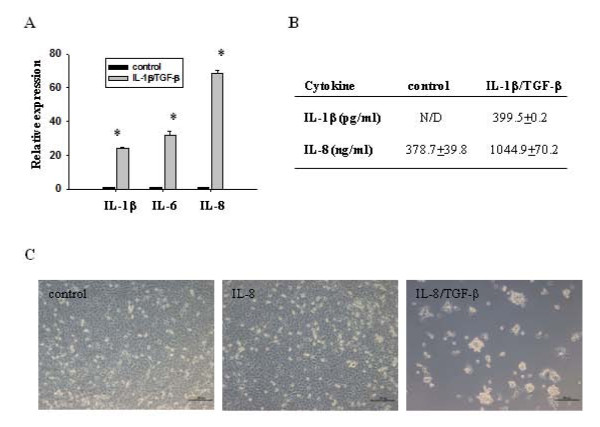
**Role of IL-1β, IL-6 and IL-8 in IL-1β/TGF-β-induced LN-229 neurosphere formation**. (**A**) Relative gene expressions of IL-1β, IL-6, and IL-8 were determined by qRT-PCR. β-actin was used as an internal normalization control. Error bars represent SEM. *P < 0.05. (**B**) Secretion of IL-1β and IL-8 from control LN-229 cells and IL-1β/TGF-β-induced neurosphere cells. The same number of LN-229 cells was cultured in SFM with or without IL-1β/TGF-β for six days, and then culture media was removed. Cells were washed with PBS, and cultured in fresh SFM without IL-1β/TGF-β for 24 hours. The media were collected, followed by ELISA analysis. N/D: not detected. (**C**) Combination of IL-8 and TGF-β induce formation of neurospheres. LN-229 cells were cultured in SFM in the presence or absence of IL-8 or IL-8/TGF-β for seven days. Scale bar = 200 μm.

To test whether other cytokines are involved in IL-1β/TGF-β-mediated induction of LN-229 neurospheres, we compared the gene expression of IL-6 and IL-8 in IL-1β/TGF-β-induced neurospheres and control cells using real time PCR. IL-1β gene expression was also examined to determine whether there was an autocrine positive feedback for IL-1β expression. It was found that mRNA levels of IL-6 and IL-8 were dramatically increased in the neurosphere cells induced by IL-1β/TGF-β, compared to that in control cells (Figure 7A). IL-1β gene expression was also up-regulated in cytokine-induced neurospheres, indicating that IL-1β can induce its own biosynthesis in LN-229 cells. Augmented expression and secretion of IL-1β and IL-8 from IL-1β/TGF-β-induced neurospheres were further confirmed using cytokine-specific ELISA (Figure 7B).

To determine whether IL-6 and IL-8 are involved in IL-1β/TGF-β-mediated induction of neurosphere formation, LN-229 cells were stimulated with IL-6 or IL-8 alone, or in combination with TGF-β in serum-free medium for seven days. Interestingly, IL-6 alone or in combination with TGF-β failed to induce neurosphere formation (data not shown). In contrast, neurospheres were induced in the presence of IL-8 and TGF-β, but not with IL-8 alone (Figure 7C). This result indicates that IL-8 signaling might be involved in IL-1β/TGF-β-mediated induction of LN-229 neurospheres. However, similar to IL-1β and TGF-β, IL-8 signaling alone is not sufficient for the induction of neurospheres from monolayer LN-229 cells.

## Discussion

Cytokines secreted either by cancer cells themselves or by the surrounding stromal cells and infiltrating leukocytes are thought to play a critical role in glioma malignancy [41]. IL-1β and TGF-β are elevated significantly in high grade gliomas and that has been associated with poor prognosis in glioma patients [42-45]. Despite recent advances in neuro-oncology, the contribution of IL-1β and TGF-β to glioma development and recurrence has not been clearly delineated. Here, we demonstrate that the combination of IL-1β and TGF-β can promote self-renewal and oncogenic capability of glioma cells by inducing expression of stemness factor genes Bmi-1, LIF and Notch-2.

In this study, we found that a human tumorigenic glioma cell line LN-229 grew as a monolayer culture and expressed nestin at a very low level under serum-free conditions. Addition of both IL-1β and TGF-β, but not each cytokine alone, to the cell cultures caused monolayer cells to form neurospheres expressing up-regulated nestin together with other stemness genes including LIF, Notch-2 and Bmi-1. The neurospheres could be induced by IL-1β/TGF-β when LN-229 cells were plated at a clonal density, and proliferated at a much slower rate than the control monolayer cells. This behavior is consistent with traits of cancer stem cells that divide more slowly than cancer cells [46]. Compared to control monolayer culture, IL-1β/TGF-β-induced neurosphere cells formed more and larger colonies in soft agar, and displayed increased chemo-resistance, invasiveness and oncogenic potential, all of which are typical characteristics of GSCs. Overall, these results indicate that IL-1β/TGF-β-induced neurospheres contain an enriched population of GSCs. This finding supports the hypothesis that IL-1β/TGF-β may promote glioma progress and recurrence by inducing self-renewal of glioma cells.

The IL-1β/TGF-β-induced LN-229 neurospheres displayed increased nestin expression (Figure 2), but were CD133^- ^(data not shown). CD133 has often been used as a GSC marker [33,47]. However, recent studies reveal that GSCs can be either CD133^+ ^or CD133^-^, but both types of GSCs express nestin in serum-free conditions and are capable of self-renewal and generate highly malignant tumors [48]. In addition, nestin expression correlates well with histological grade of glioma and clinical outcome [49]. Thus, nestin seems to be a more critical marker for identification of GSCs.

LIF is a neuropoietic cytokine that stimulates self-renewal capacity, prevents differentiation of NSCs and GSCs *in vitro *and *in vivo *[31,50], and mediates TGF-β-induced GSC self-renewal [31]. Up-regulation of LIF expression in IL-1β/TGF-β-induced neurosphere cells (Figure 2) supports that cytokine-induced neurosphere cells are developed through self-renewal. However, continued up-regulation of LIF expression may not be required for the maintenance of stemness as its expression in passage 2 of the neurosphere cells in the absence of IL-1β and TGF-β is similar to that in control LN-229 cells (Figure 6).

Notch signaling is required for GSC proliferation and blockade of the Notch pathway can inhibit the growth of tumor neurospheres and xenografts [51,52]. Among the four Notch paralogs, Notch-2 is highly expressed in glioblastomas and may have a predominant role in glioblastoma cell growth [52,53]. Our studies have shown that Notch-2, but not Notch-1 or Notch-3 (data not shown), is up-regulated in IL-1β/TGF-β-induced neurosphere LN-229 cells. This observation is consistent with the thought that Notch-2 may play a major role in glioma malignancy. Bmi-1, a transcriptional repressor belonging to the polycomb group protein family, is essential for the proliferation and self-renewal of NSCs and GSCs [54,55]. Overexpression of Bmi-1 can induce astrocytes into NSC-like cells under serum-free condition [56]. Thus, the augmented expression of Bmi-1 in IL-1β/TGF-β-induced neurosphere LN-229 cells favors the hypothesis that Bmi-1 plays a critical role in inducing monolayer LN-229 cells to display a GSC phenotype.

The role of Bmi-1 in GSC self-renewal and oncogenic potential is further highlighted in cytokine-withdrawn LN-229 cells. In the absence of IL-1β/TGF-β, dissociated IL-1β/TGF-β-induced neurosphere cells proliferated as a mix of monolayer and cluster cells, suggesting a reduced self-renewal capability. Furthermore, the cytokine-withdrawn cells maintained strong oncogenic potential with a similar colony number as the cytokine-induced neurosphere cells in a soft agar assay, but with a slightly reduced neurosphere size. Gene expression analysis showed that transcripts of nestin and LIF in cytokine-withdrawn cells were reduced to the control level. Notch-2 expression was also dramatically decreased compared to that in cytokine-induced neurospheres, but it was still significantly higher than that in control LN-229 cells. Remarkably, Bmi-1 levels did not decrease after cytokine withdrawal. The reduced expression of LIF and Notch-2 and sustained Bmi-1 expression in cytokine-withdrawn cells are consistent with the observation that these cells display a mixed phenotype of monolayer and cluster cells and maintain a relatively strong oncogenic potential. Our data support the notion that Bmi-1 is critical for maintenance of GSC self-renewal. However, we cannot exclude the possibility that other unidentified factors are also involved in the sustained clonogenicity of cytokine-withdrawn cells. Future studies are planned to investigate the molecular mechanisms of persistent expression of Bmi-1 and Notch-2 in IL-1β/TGF-β-induced neurosphere cells upon cytokine withdrawal.

Our data suggest that there is a close association between increased expression of SIP1, N-cadherin and β1-integrin and stronger invasiveness of the cytokine-induced neurospheres. It has been reported that β1-integrin is over-expressed in several types of gliomas and blocking β1-integrin inhibits the motility and invasion of glioma cells [57,58]. Similarly, N-cadherin, a calcium-binding membrane glycoprotein that mediates cell-cell adhesion, is up-regulated in malignant gliomas cells compared to normal brain tissue [59], and plays a critical role in glioma cell migration [60-62]. Reduction of SIP1, a member of the ZEB family of transcription factors, has been linked to the impairment of colony formation in soft agar, migration and invasion of tumorigenic glioma cells [63]; while overexpression of SIP1 in nontumorigenic glioma cells enhances their colony formation in soft agar, migration and invasion.

In addition, IL-1β, IL-6 and IL-8 and their cognate receptors are also elevated in malignant gliomas [64,65]. Our studies have shown that IL-1β can also induce the production of IL-6 and IL-8 in LN-229 neurospheres. This result is consistent with previous reports that IL-1β can induce the expression and secretion of IL-6 and IL-8 in human gliomas [41,65,66]. However, it seems that only IL-8, but not IL-6, is involved in IL-1β/TGF-β-induced neurosphere formation from monolayer LN-229 cells. That is, only the combination of IL-8 and TGF-β, but not the combination of IL-6 and TGF-β, can induce neurospheres from LN-229 cells cultured in serum-free medium. Nonetheless, it remains to be determined whether IL-6 and IL-8 participate in the IL-1β/TGF-β-induced self-renewal of LN-229 GSCs.

## Conclusions

In summary, we have used a human LN-229 glioma cell line to examine the effect of IL-1β/TGF-β signaling on induction of self-renewal and oncogenic capability of glioma cells. Recent studies have focused on a small population of GSCs, which are capable of self-renewal, initiation of neurosphere growth and generation of malignant tumors, for development of therapeutic strategies [12]. However, current standard *in vitro *conditions to define GSCs lack cytokines that are highly present in the glioblastoma microenvironment. Our findings indicate that glioma cells that fail to self-renew to generate neurospheres in standard stem cell-enrichment conditions can gain this ability after exposure to IL-1β and TGF-β. Thus, targeting GSC-promoting microenvironment cytokines or interference with Bmi-1 may be essential for a higher efficacy of glioma therapies. Future studies aimed to determine the molecular mechanisms of IL-1β/TGF-β-mediated induction of self-renewal may lead to the invention of novel therapies that target the process of GSC activation.

## Abbreviations

bFGF: basic fibroblast growth factor; CSCs: cancer stem cells; EGF: epidermal growth factor; GSC: glioma stem cell; IL-1β: interleukin-1 beta; NSC: neural stem cells; SFM: serum-free medium; TGF-β: transforming growth factor-beta; TICs: tumor-initiating cells.

## Competing interests

The authors declare that they have no competing interests.

## Authors' contributions

LW, ZL, SBalivada, TS, SBossman, JS and DT designed the experiments. LW, ZL, S Balivada, TS, LP and MP performed the experiments. LW, JS, SBossman and DT wrote the manuscript. All authors were involved in revising the manuscript and have given final approval of the version to be published.
